# Sodium Valproate-Induced Hyperammonemia: A Case Series in a Tertiary Care Hospital

**DOI:** 10.7759/cureus.65390

**Published:** 2024-07-25

**Authors:** Pallavi Abhilasha, Neena Bhatti, Girish Joseph, Dinesh K Badyal

**Affiliations:** 1 Psychiatry, Christian Medical College and Hospital, Ludhiana, IND; 2 Pharmacology, Christian Medical College and Hospital, Ludhiana, IND

**Keywords:** hyperammonemic encephalopathy, anti-convulsant, adverse effects, hyperammonemia, sodium valproate

## Abstract

Background: Sodium valproate (VPA) is an extensively used anti-convulsant, which is an effective drug for treatment of epilepsy in adults and children, as well as for conditions like migraine, bipolar disorder, mania, and trigeminal neuralgia. Sedation, vertigo, ataxia, dose-dependent tremors, headaches, and gastrointestinal side effects are the most often reported adverse effects associated with VPA. A potential life-threatening event reported with VPA is hyperammonemia (HA), which is defined as an increase in serum level of ammonia. Only 587 reported cases of HA were found in the VigiAccess database, representing a mere 0.6% of the 95,000 reported adverse events linked to VPA. Hence, this case series was conducted with emphasis on monitoring of increased serum ammonia levels with or without hepatic enzymes increase for patients who are on VPA.

Aims and objectives: To assess elevated serum ammonia levels following VPA administration, and to ascertain the percentage of individuals with hepatic enzymes increased who took VPA and subsequently had elevated serum ammonia levels.

Methods: This study was conducted at the adverse drug reaction (ADR) monitoring centre (AMC) of the Pharmacovigilance Programme of India (PvPI) and Department of Psychiatry, Christian Medical College and Hospital (CMC&H), Ludhiana. The study comprised of 12 patients who were exclusively on VPA and exhibited symptoms related to elevated serum ammonia. An informed consent form (ICF) was provided to the patient prior to taking their personal details. Laboratory investigations were done to establish the diagnosis and liver function tests (LFTs), chiefly ALT (alanine transferase) and AST (aspartate aminotransferase) were also performed. It is a descriptive study which was for a time period of six months.

Results: This study includes 12 patients who had HA confirmed by laboratory investigation. Out of these 12 patients, two patients (17%) had a corresponding increase in LFT. The average as of the patients was 53.08 years and average serum ammonia levels were 219.15. None of the patients who presented with HA progressed to hyperammonemic encephalopathy (HAE).

Conclusion: This case series on valproate-induced HA should be of interest to psychiatrists, physicians, internists, family medicine physicians, hospitalists, and surgeons who will have patients on VPA. Delay in recognition of HA can result in the development of potentially life-threatening complications. Rapid diagnosis and management will help in reducing the number of cases which progress to encephalopathy which is highly fatal.

## Introduction

World Health Organization (WHO) defines adverse drug reaction (ADR) as “a response to a medication that is noxious and unintended and occurs at doses normally used in man” [1]. ADRs have a negative effect on patients' quality of life in addition to placing more strain on the healthcare system. They are posing a serious threat to public health owing to increasing morbidity and mortality [2]. Sodium valproate (VPA) is an extensively used anti-convulsant which is an effective drug for treatment of epilepsy in adults and children, as well as for conditions like migraine, bipolar disorder, mania and trigeminal neuralgia [3].

VPA functions primarily as an inhibitor of gamma-aminobutyric acid (GABA) transaminase, a voltage-gated sodium channel blocker, and a T-type calcium channel blocker [4]. The action of onset is fast as patient starts responding to the drug within three to five days. The dose of VPA comprises of strengths of 125 mg, 250 mg, and 500 mg concentrations as well as a 250 mg and 500 mg extended-release formulation [5]. VPA is usually a safe drug with a wide therapeutic index ranging from 50-125 mcg/ml [6]. Sedation, vertigo, ataxia, dose-dependent tremors, headaches, and gastrointestinal side effects, such as weight gain, stomach discomfort, nausea, vomiting, diarrhea, constipation, and decreased appetite are the most often reported adverse effects associated with VPA [7]. 

Another potential life-threatening event reported with VPA is hyperammonemia (HA), which is defined as an increase in serum level of ammonia. The occurrence of HA is reported to be one in 20,000 patients. Measuring the serum ammonia levels is crucial because they can cause hyperammonemic encephalopathy (HAE), which can occur with or without an increase in liver function tests (LFTs) [6]. Hepatic HA damages multiple organs, induces inflammation, and has neurotoxic effects and is associated with liver cirrhosis. Whereas on the other hand, the etiology of non-hepatic HA encompasses organ transplantation, hematologic malignancy, protein load, and enhanced catabolism [8]. Patients with non-hepatic HAE have an exceptionally high risk of morbidity and fatality. The most frequent manifestation in these individuals is confusion, which is characterized by apathy, sleepiness and diminished cognitive function, followed by coma and death [6,8].

Only 587 reported cases of HA were found in the VigiAccess database, representing a mere 0.6% of the 95,000 reported adverse events linked to VPA [9]. It is crucial to monitor elevated serum ammonia levels linked to VPA because of the significant morbidity and mortality associated with HAE, which can arise from elevated serum ammonia levels. Since there is limited information on this topic, this case series emphasis on monitoring of increased serum ammonia levels with or without hepatic enzymes increase for patients who are on VPA.

## Materials and methods

This study was conducted at the ADR monitoring centre (AMC) of the Pharmacovigilance Programme of India (PvPI) and Department of Psychiatry, Christian Medical College and Hospital (CMC&H), Ludhiana. A total of 12 diagnosed cases with increased serum ammonia were reported from the Department of Psychiatry at CMC&H, Ludhiana, from August 2023 to February 2024. The serum ammonia was measured in the suspected patients on presenting to the hospital. When they started recovering from the symptoms after receiving treatment, the serum ammonia was tested again after a period of three to four days. No follow-up was required as all the patients were either recovered or recovering. An informed consent form (ICF) was provided to the patient prior to taking his personal details. The ICF was in two languages: English and was also translated to Hindi (which is a regional language). The format of ICF is shown in Annexure I.

The causality assessment was done by applying the Naranjo causality assessment scale. The Adverse Drug Reaction (ADR) Probability Scale was developed in 1991 by Naranjo. It consists of a series of 10 questions with responses of “yes”, “no” and “don’t know” where different point values (-1, 0, +1, or +2) are assigned to each answer. The reaction is deemed definite if the score is 9 or above, probable if it is 5 to 8, possible if it is 1 to 4, and doubtful if it is 0 or lower. The total scores range from -4 to +13 [10]. The details of the Naranjo probability scale is shown in Annexure II.

Study design

This was a descriptive case study. 

Inclusion criteria

Patients diagnosed with increased serum ammonia after consuming VPA were considered.

Exclusion criteria

1. Patients having leukemia, underlying hepatic disease, malignancy, infections or any other medical conditions which predispose to increased serum ammonia were excluded from the study.

2. Patients on any other concomitant psychotropic medications were excluded from the study.

Study procedure

The study comprised of patients who were on VPA and exhibited symptoms related to elevated serum ammonia. Laboratory investigations were done to establish the diagnosis and LFTs, chiefly ALT and AST were also performed. The AMC was informed and the details of these patients were collected. The patients were provided with ICF who agreed to participate in the study. The study did not require approval from research and ethical committee as it was a case series.

Statistical analysis

The results were analyzed using descriptive statistics and presented as percentage and proportions. Other than this, a case series does not have any control group, no further statistics are required for analysis.

## Results

A total of 12 cases of VPA induced HA were collected from the Psychiatry Department of CMC&H, Ludhiana, within a span of six months. The most common symptoms of HA observed in these patients included aggressive and violent behaviour, mental confusion, irritability, slurring of speech, and coordination and balance disorders.

Age

The age of patient ranged from 37-67 years. Majority of the patients (42%) were in the each age group of 36-45 years and 55-65 years, thereby indicating no age preponderance for HA. This is shown in Table 1.

**Table 1:** Baseline characteristics

Characteristic	Number	Units
Average age	53.08	Years
Average serum ammonia	219.15	ug/dl

Gender

The incidence of HA was higher in males (10 out of 12 patients),which is 83% as compared to females (two out of 12 patient) which is 17%.

Serum ammonia level

The normal serum ammonia levels vary from 15-45 ug/dl. However, in this study the average serum ammonia of the 12 patients is significantly higher at 219.15 ug/dl. This is shown in Table 1.

Comprehensive patient review

All the patients had a Naranjo score ranging from 5-7, which indicates a probable relationship with the suspect product. A detailed overview of all these patients is shown in Table 2.

**Table 2:** Comprehensive patient overview

Pt.no	Age	Gender	Indication	Duration since on valproate	Temporal association	De-challenge	Causality assessment using Naranjo scoring	Outcome
1	53	M	Bipolar disorder	3 months	Strong	+ve	Probable	Resolving
2	60	M	Bipolar disorder	1 month	Strong	+ve	Probable	Resolving
3	44	M	Bipolar disorder	3 weeks	Strong	+ve	Probable	Resolved
4	58	M	Affective disorder	2.5 months	Strong	+ve	Probable	Resolving
5	65	M	Bipolar disorder	2 weeks	Strong	+ve	Probable	Resolving
6	64	M	Altered sensorium	5 months	Strong	+ve	Probable	Resolved
7	44	F	Bipolar disorder	1.5 months	Strong	+ve	Probable	Resolving
8	41	F	Bipolar disorder	3 months	Strong	+ve	Probable	Resolving
9	67	M	Bipolar affective disorder	1 week	Strong	+ve	Probable	Resolving
10	40	M	Bipolar affective disorder	2 months	Strong	+ve	Probable	Resolving
11	64	M	Bipolar disorder	2 weeks	Strong	+ve	Probable	Resolving
12	37	M	Bipolar disorder	3 months	Strong	+ve	Probable	Resolving

Laboratory investigations

Out of 12 patients, only two patients (17%) had increased SGOT and SGPT in response to increased serum ammonia. Thus, 83% of the patients were at risk of progressing to non-hepatic encephalopathy owing to increased ammonia.

Table 3 denotes detailed laboratory investigations of the patients.

**Table 3:** Detailed laboratory investigations of the patients SGOT: Serum glutamic-oxaloacetic transaminase; SGPT: Serum glutamic pyruvic transaminase

Pt.no	Serum ammonia (ug/dl)	SGPT (u/L)	SGPT (u/L)
1	449	23.5	30.8
2	161	30.2	45.7
3	155	28.8	40.8
4	219.8	26.9	13.2
5	280	136.9	120.2
6	408	22.9	16.2
7	135	25.5	26.8
8	130	89.3	90.4
9	127.1	20.7	34.4
10	117	29.7	33.4
11	225	17.9	27.9
12	223	30.3	35.5

## Discussion

VPA is used for a number of mental and neurological conditions. It can be used in conjunction with anti-psychotics to treat schizophrenia and schizoaffective disorders, even though it’s main indication is mood disorders [6]. VPA induced HA is a rare adverse effect which occurs even at therapeutic drug levels [11]. It is a challenge to identify HA symptoms in psychiatric patients as the clinical features of HA are similar to what are seen in psychiatric patients. This challenge can be overcome by identifying early VPA induced HA by closely monitoring the patient for mental status changes, focal neurological impairments, disorientation and delirium. The patient should have their drug and ammonia levels tested right away if they experience any neurological side effects or drowsiness [12].

Although the exact mechanism of HA is unknown, it is postulated that there is a genetic flaw in either ornithine transcarbamylase (OTC) or CPS, two enzymes involved in the urea cycle's early stages, which ultimately disrupts the urea cycle. This disruption of urea cycle results in accumulation of ammonia which is detrimental of the central nervous system (CNS). The CNS utilizes glutamine synthetase, which is found in astrocytes, to convert ammonia to glutamine. Consequently increased concentration of ammonia causes glutamine levels to rise, which in turn causes astrocyte enlargement and cerebral edema [7]. Thus, it is reasonable to assume that individuals with urea cycle disorders are unquestionably more likely to have HA [13].

In this study, the percentage of males (83%) having HA as compared to females (17%) is significantly higher, which is at odds with what is usually observed as females have been known to have a higher incidence of HA as compared to males [14]. This could be possible considering an unequal distribution of patients who were put of valproate therapy. However, a study conducted in 2020, reported a higher male preponderance to HA as compared to females which was again owing to more number of males in the study [15]. In this study, the average age was 53.08 years, which is in concordance to another study where the average age was 52 years [16]. All the patients with HA were asymptomatic and had a benign course and quickly recovered after lactulose therapy was used to lower blood ammonia.

VPA induced HA may present as symptoms ranging from subtle behaviour changes to fatal valproate induced encephalopathy. It is critical to be vigilant in the detection of encephalopathy signs. There are various therapeutic options available to patients diagnosed with VPA induced HA. The cornerstone of management is withholding valproate and providing supportive care for encephalopathy caused by valproate. Catabolism is to be stopped in order to lower the amount of ammonia produced. A non-absorbable disaccharide such as lactulose which lowers the production and absorption of ammonia is routinely used. In addition to this, supplementing with carnitine lowers elevated ammonia levels by binding to VPA and disabling the urea cycle. Limiting protein intake and using L-carnitine can be beneficial. Haemodialysis is recommended when the ammonia level is more than four times the upper limit of normal Other treatment options include use of mannitol, vitamin B, rifaximin, intravenous hydration and vitamin B [17,18].

It has been determined that HA is an uncommon but noteworthy adverse effect of using VPA which carries a high risk of death [19]. Therefore, this case series highlights the importance of monitoring serum ammonia levels in patients who have been started on VPA. In this study, all the patients were diagnosed cases of bipolar disorder on VPA.

The current study is limited by the number of patients. An independent study can be done on VA induced HA, where the serum ammonia levels of the patients would be followed serially and also the response of serum ammonia level to different treatment modalities. Moreover, the current study is focused on finding VPA induced HA cases as a part of initiative of the AMC of the institute. Further research is therefore warranted on deciphering the etiology primarily on non-hepatic encephalopathy. This can be achieved by regular monitoring of ammonia levels of patients on VPA, which can be logistically challenging and resource-intensive. In addition to this, in this study only patients on VPA were included in the study, so no role of any similar psychotropic medication causing HA was explored.

## Conclusions

Valproate-induced HA requires early diagnosis and management. Focused research efforts in understanding the condition will help decrease its incidence. This case series should be of interest to psychiatrists, physicians, internists, family medicine physicians, hospitalists, and surgeons who will have patients on VPA. Delay in recognition of HA can result in the development of potentially life-threatening complications. Since VPA frequently causes a modest rise in plasma ammonia levels which is asymptomatic, it is important to recognize the symptoms of HA promptly. Rapid diagnosis and management will help in reducing the number of cases, which progress to encephalopathy which is highly fatal.

## Figures and Tables

**Figure 1:**
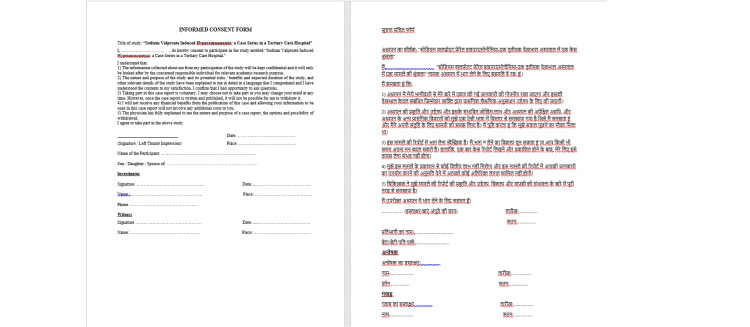
Depicts the ICF in English and Hindi for the patients ICF: Informed consent form

**Figure 2:**
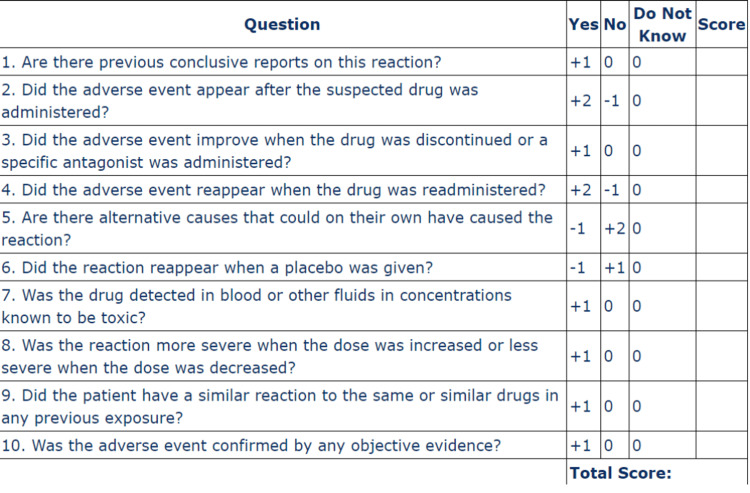
Naranjo causality assessment scale [10]

## Appendices

Annexure I

This shows the ICF administered to patients in both English and the regional language Hindi.

Annexure II

This annexure shows the Naranjo scoring using a series of 10 questionnaires.  

## References

[1] Siddhu CK, Joseph G, Bhatti N, Badyal D (2023). Paracetamol induced facial puffiness: an uncommon case report. Nat J Pharm Therap.

[2] Khalil H, Huang C (2020). Adverse drug reactions in primary care: a scoping review. BMC Health Serv Res.

[3] Zhang X, Lu W, Mo X (2023). Sodium valproate-induced nocturnal enuresis in epilepsy: Three case reports and a review of the literature. Front Neurol.

[4] Mello ML (2021). Sodium valproate-induced chromatin remodeling. Front Cell Dev Biol.

[5] Rahman M, Awosika AO, Nguyen H (2024). Valproic acid. https://www.ncbi.nlm.nih.gov/books/NBK559112/.

[6] Farooq F, Sahib Din J, Khan AM, Naqvi S, Shagufta S, Mohit A (2017). Valproate-induced hyperammonemic encephalopathy. Cureus.

[7] Shah S, Wang R, Vieux U (2020). Valproate-induced hyperammonemic encephalopathy: a case report. J Med Case Rep.

[8] Kim JH, Jeon H, Lee SS (2021). Impact of non-hepatic hyperammonemia on mortality in intensive care unit patients: a retrospective cohort study. Korean J Intern Med.

[9] (2024). VigiAccess. https://www.vigiaccess.org/.

[10] (2024). LiverTox: clinical and research information on drug-induced liver injury. https://www.ncbi.nlm.nih.gov/books/NBK548069/.

[11] Hosseini H, Shafie M, Shakiba A, Ghayyem H, Mayeli M, Hassani M, Aghamollaii V (2023). Valproic acid-induced hyperammonemia in neuropsychiatric disorders: a 2-year clinical survey. Psychopharmacol (Berl).

[12] Cofini M, Quadrozzi F, Favoriti P, Favoriti M, Cofini G (2015). Valproic acid-induced acute pancreatitis in pediatric age: case series and review of literature. G Chir.

[13] Palomino Pérez LM, Martín-Rivada Á, Cañedo Villaroya E, García-Peñas JJ, Cuervas-Mons Vendrell M, Pedrón-Giner C (2020). Use of carglumic acid in valproate-induced hyperammonemia: 25 pediatric cases. JIMD Rep.

[14] McMorris T, Chu A, Vu L, Bernardini A (2021). Hyperammonemia in patients receiving valproic acid in the hospital setting: a retrospective review. Ment Health Clin.

[15] Tseng YL, Huang CR, Lin CH (2014). Risk factors of hyperammonemia in patients with epilepsy under valproic acid therapy. Medicine (Baltimore).

[16] Jacoby KJ, Singh P, Prekker ME, Leatherman JW (2020). Characteristics and outcomes of critically ill patients with severe hyperammonemia. J Crit Care.

[17] Qayyum W, Yousafzai ZA, Iqbal MS, Khan S, Amin QK, Shafiqs I (2022). Valproate induced hyperammonemic encephalopathy. J Ayub Med Coll Abbottabad.

[18] Lewis C, Deshpande A, Tesar GE, Dale R (2012). Valproate-induced hyperammonemic encephalopathy: a brief review. Curr Med Res Opin.

[19] Camilleri L (2018). Lesson of the month 1: Sodium valproate-induced encephalopathy. Clin Med (Lond).

